# Association between serum uric acid and colorectal cancer risk in European population: a two-sample Mendelian randomization study

**DOI:** 10.3389/fonc.2024.1394320

**Published:** 2024-07-01

**Authors:** Jinsong Zhou, Rong Fu, Juwei Zhang, Suhong Zhang, Zhifeng Lin, Zheng Lin, Xin Liu, Xiaolu Xu, Yulun Chen, Zhijian Hu

**Affiliations:** Department of Epidemiology and Health Statistics, Fujian Provincial Key Laboratory of Environment Factors and Cancer, School of Public Health, Fujian Medical University, Fuzhou, China

**Keywords:** uric acid, colorectal cancer, Mendelian randomization, causal association, meta-analysis

## Abstract

**Objectives:**

This study aimed to explore the potential causal associations between serum uric acid (SUA) and the risk of colorectal cancer, colon cancer and rectal cancer.

**Methods:**

Twenty-six SUA-related single nucleotide polymorphisms which were identified by a large meta-analysis of genome-wide association studies (GWASs) were used as instrumental variables in the two-sample Mendelian randomization (MR) study. Meta-analyses were used to synthesize the results of multiple GWASs which were extracted from the MRC Integrative Epidemiology Unit GWAS database for each type of cancer. The inverse variance weighted (IVW) method was used as the primary MR method to analyze the association between SUA and colorectal cancer risk. Several sensitivity analyses were performed to test the robustness of results.

**Results:**

The IVW method showed that there were no causal relationships between SUA and the risk of colorectal cancer [odds ratio (OR): 1.0015; 95% confidence interval (CI): 0.9975–1.0056] and colon cancer (OR: 1.0015; 95% CI: 0.9974–1.0055). The SUA levels were negative correlated with rectal cancer risk (OR: 0.9984; 95% CI: 0.9971–0.9998). The similar results were observed in both males (OR: 0.9987; 95% CI: 0.9975–0.9998) and females (OR: 0.9985; 95% CI: 0.9971–0.9999). The sensitivity analyses suggested no evidence of heterogeneity or horizontal pleiotropy. The leave-one-out analyses showed that one SNP (rs1471633) significantly drove the causal effect of SUA on rectal cancer risk. The MR-Egger regression and weighted median both showed that there were no causal relationships between SUA and the risk of colorectal cancer and its subtypes.

**Conclusion:**

Overall, there was no linear causal association between SUA and the risk of colorectal cancer. However, further research is needed to investigate the role of higher SUA levels such as hyperuricemia or gout in the occurrence of colorectal cancer.

## Introduction

Colorectal cancer is the world’s second deadly cancer with 1.93 million new cases and 903,859 deaths in 2022. The incidence and mortality of colorectal cancer increase with age and are higher in males than females (1). The identification of pathogenic factors is very important for the prevention and control of colorectal cancer, but the factors driving the increasing incidence and mortality are still largely unknown. Established nonmodifiable risk factors of colorectal cancer are age, male sex and family history, and the modifiable risk factors are smoking, alcohol use, obesity and red meat (2). The nonmodifiable risk factors are generally immutable, so it is necessary to explore whether there are potentially modifiable risk factors for colorectal cancer.

Serum uric acid (SUA) is the final oxidation product of purine nucleotide degradation. Some physiological or pathological reactions will cause the imbalance of SUA metabolism, resulting in hyperuricemia (3, 4). Hyperuricemia is a chronic progressive disease without obvious clinical symptoms and is associated with gout, cardiovascular diseases, metabolic syndrome and kidney diseases (5). Many studies have also explored the associations between elevated SUA levels and cancer risk, but the conclusions were inconsistent. SUA as an antioxidant with beneficial effects has been reported a protective role against cancer (6, 7). A systematic review of prospective studies reported that elevated SUA levels had no statistically significant association with overall cancer risk and results for specific cancers were limited and mainly negative (8). Mounting evidence indicated that high SUA levels represent a risk factor in various cancers through generating inflammatory reactions and oxidative stress (9, 10). For colorectal cancer, a population-based prospective cohort study found no significant association between SUA and colorectal cancer risk (11). A large Swedish cohort study reported that elevated SUA levels were positively associated with the risk of colorectal cancer in males (12). The similar results were also found in Chinese males (13). Another cohort study using the UK Biobank database found that SUA showed a U-shaped association with colon cancer risk in females and males with SUA ≤ 3.5 mg/dL and females with SUA > 8.4 mg/dL had a higher risk of rectal cancer than those with SUA between 4.4 and 5.4 mg/dL (14). The above controversial conclusions might be attributed to potential confounding factors and reverse causation, which were the limitations of observational studies.

Mendelian randomization (MR) is a method that uses genetic variants as instrumental variables to analyze the unbiased causal effects of exposures and outcomes. Since the distribution of genetic variants is random and genotype is not influenced by diseases, it can avoid some limitations of observational studies (15). Kobylecki et al. used urate solute carrier family 2 member 9 rs7442295 genotype as an instrumental variable and found that high plasma urate levels were genetically associated with high cancer incidence and high all-cause mortality (16). There were potential causal associations between higher SUA levels and prostate cancer risk in East Asian population (17). However, the results of a one-sample MR study from the UK Biobank database did not support a causal relationship between SUA and lung cancer (18). Jiang et al. also did MR analysis to identify the causal effect of SUA on eight site-specific cancers risk and a total of six MR methods all showed no significant causality (19). However, it was unknown whether SUA was causally associated with colorectal cancer risk.

In this study, a two-sample MR study was conducted to explore the potential causal relationships between SUA and colorectal cancer risk and its subtypes with 26 single nucleotide polymorphisms (SNPs) as instrumental variables. The findings would provide additional evidences for the causal effect of SUA on the colorectal cancer risk.

## Methods

### Study design

**Figure 1:**
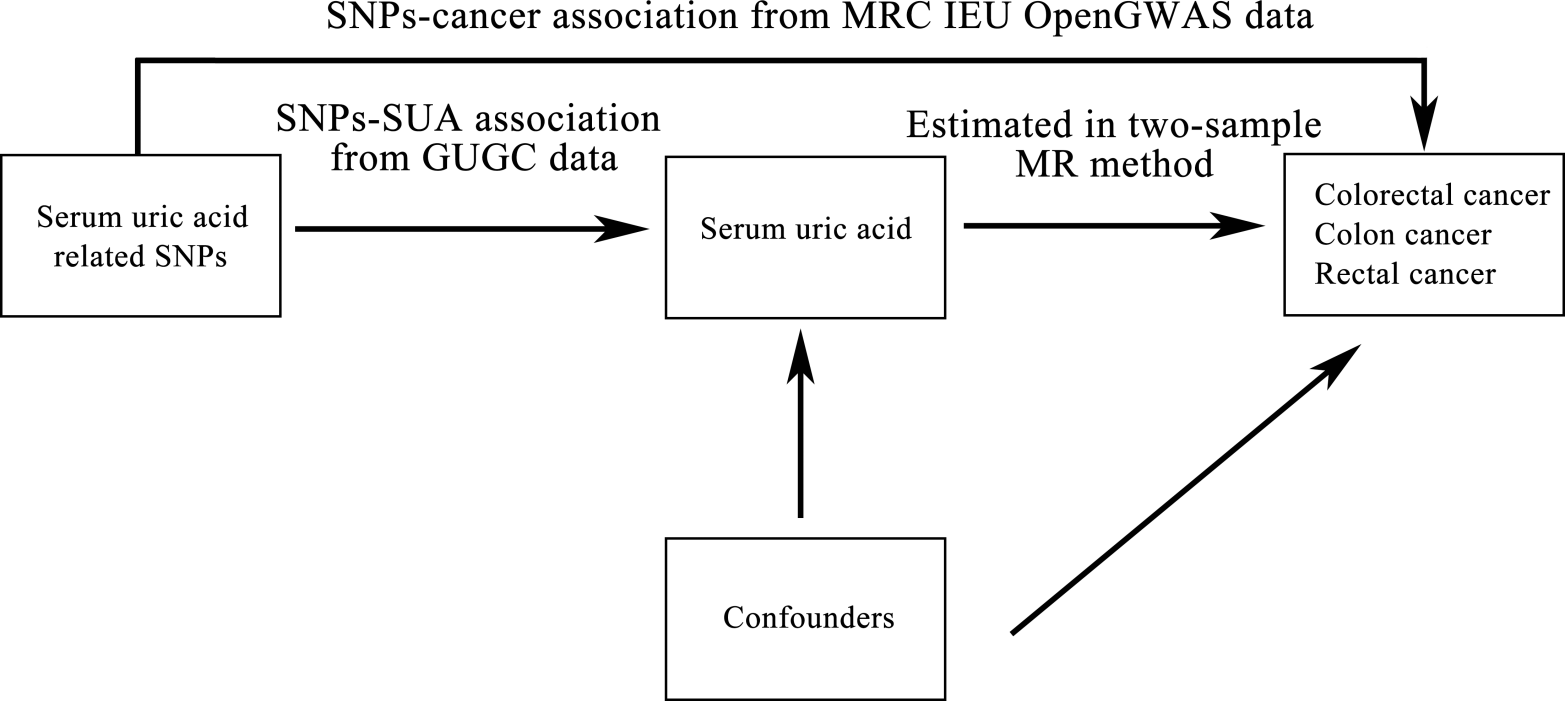
Diagram of two-sample Mendelian randomization analysis of serum uric acid levels and cancer risk. SNP: single nucleotide polymorphism; SUA: serum uric acid; GUGC: Global Urate Genetics Consortium; MR: Mendelian randomization; IEU: Integrative Epidemiology Unit; GWAS: genome-wide association study.

The causal relationships between SUA and the risk of colorectal cancer, colon cancer and rectal cancer were inferred using two-sample MR study with SUA associated SNPs as instrumental variables. **Figure 1** shows the diagram of two-sample MR analysis of SUA and cancer risk. Three core assumptions should be satisfied to guarantee MR effect estimates without bias. First, the SNPs should have a strong association with SUA. Second, the SNPs should have been independent of confounding. Third, the SNPs should have affected cancer risk only via SUA and could not have a direct association.

### Genetic instruments for SUA

The SNPs-SUA sample was extracted from a large meta-analysis of 48 genome-wide association studies (GWASs) of 110,347 European individuals based on Global Urate Genetics Consortium (GUGC) data. In each GWAS, genotype imputation was carried out using the HapMap 2 data as the reference (20). The mean (standard deviation) of SUA levels in these studies ranged from 3.9 to 6.1 mg/dL (0.92 to 1.68 mg/dL) (**Supplementary Figure S1**). To satisfy the MR assumptions, we restricted the instrumental SNPs to those highly associated with SUA levels (P<5.00E-8) and used a conservative clumping threshold with r^2^<0.001 to ensure linkage equilibrium. In addition, F-statistic value was used to assess the strength of each SNP. The SNP with F-statistic value larger than 10 was considered to have a strong potential to predict SUA levels. Finally, a total of 26 SNPs which explained 7.0% of the variance in SUA levels were screened to perform the MR analysis. The overall population and gender-specific effects for SNPs-SUA associations are displayed in **Supplementary Tables S1** and **S2**, respectively.

### Genetic association with outcomes

The summary results of colorectal cancer and its subtypes were extracted from the latest MRC Integrative Epidemiology Unit (IEU) (University of Bristol) GWAS database (https://gwas.mrcieu.ac.uk/, Accessed 05 August 2023). According to the phenotypes of GWASs, they were classified as colorectal cancer, colon cancer, and rectal cancer-associated GWASs. Because there were several GWASs for each cancer, meta-analyses with random effect models were applied to synthesize the results of these GWASs.

### Statistical analysis

The fixed-effect inverse variance weighted (IVW) method was used as the primary MR method to derive an overall weighted estimate of the potential causal effect of SUA levels on cancer risk. This method assumed that all SNPs were valid instruments and combined individual MR estimates across SNPs. The same analyses were conducted in overall population, males and females, respectively. In order to test the robustness of results, several sensitivity analyses were conducted. First, the significant heterogeneity indicates the presence of horizontal pleiotropy that instrumental SNPs affect the outcome not through the exposure of interest. Therefore, Cochran’s Q test was used to assess the heterogeneity among individual SNP estimates and horizontal pleiotropy was further examined using MR-Egger regression. Second, the leave-one-out analysis was conducted to investigate the possibility that the causal association was driven by a single SNP. In addition, the MR-Egger regression and weighted median method were implemented as complements to the IVW method. The MR-Egger regression estimated the causal effects in the presence of pleiotropic effects and the weighted median method could robustly assess the causal effects even if over half of the weight came from invalid instruments.

All statistical analyses were performed using R software (version 4.1.2) with “meta” package (version 6.10) for meta-analyses, “ggplot2” package (version 3.4.1) for forest plots and “TwoSampleMR” package (version 0.5.6) for MR analyses. Two-tail P<0.05 was considered statistically significant.

## Results

### Association between SNP and cancer risk

**Figure 2:**
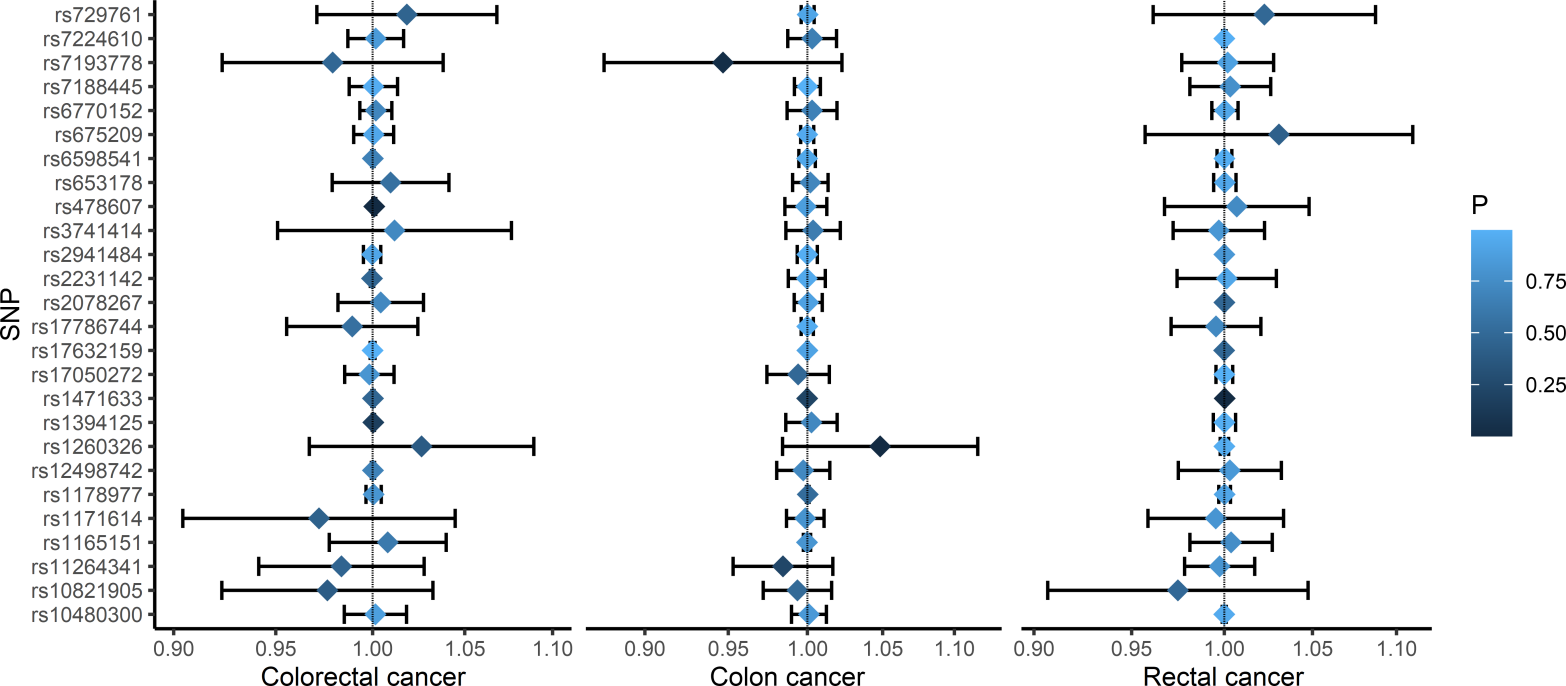
Forest plots of the associations between SNPs and the risk of colorectal cancer, colon cancer, and rectal cancer. SNP: single nucleotide polymorphism.

This study included ten cancer-related GWASs containing two colorectal cancer studies (8,679 cases and 543,000 controls), four colon cancer studies (7,862 cases and 1,0657,37 controls) and four rectal cancer studies (4,803 cases and 1,457,495 controls). Details of the GWASs used in this study are shown in **Table 1**. Meta-analyses showed that none of the 26 SUA associated SNPs was significantly related to the risk of colorectal cancer, colon cancer and rectal cancer (**Supplementary Figures S2**–**S4**). The association between each SNP and cancer risk from the meta-analyses was integrated in **Figure 2**. All the 26 SNPs were used as the instrumental variables in the MR analyses for SUA-colorectal cancer and its subtypes.

**Table 1:** Characteristics of exposure dataset and outcome datasets.

Phenotype	Consortium	Cases	Controls	Sample size	Population
Serum uric acid	GUGC			110347	European
Colorectal cancer		8679	543000	551679	European
Ieu-b-4965	UK Biobank	5657	372016	377673	European
Finn-b-C3_COLORECTAL_EXALLC	FinnGen	3022	170984	174006	European
Colon cancer		7862	1065737	1073599	European
Ukb-d-C3_COLON	Neale lab	2437	358757	361194	European
Ukb-d-C18	Neale lab	2226	358968	361194	European
Finn-b-C3_COLON_EXALLC	FinnGen	1803	174006	175809	European
Finn-b-C3_COLON_ADENO_EXALLC	FinnGen	1396	174006	175402	European
Rectal cancer		4803	1457495	1461128	European
Ukb-b-19425	MRC-IEU	1085	461925	463010	European
Ukb-b-1251	MRC-IEU	1470	461540	463010	European
Ukb-d-C_RECTUM	Neale lab	1170	360024	360024	European
Finn-b-C3_RECTUM_EXALLC	FinnGen	1078	174,006	175084	European

GUGC, Global Urate Genetics Consortium; MRC-IEU, MRC Integrative Epidemiology Unit.

### Association between SUA and cancer risk

There was no association between SUA and colorectal cancer risk [odds ratio (OR): 1.0015; 95% confidence interval (CI): 0.9975, 1.0056]. The similar result was also found in males (OR:0.9989; 95%CI: 0.9968, 1.0009) and females (OR:0.9992; 95%CI: 0.9976, 1.0009). Similarly, the SUA levels were not significantly associated with colon cancer in overall population [(OR (95% CI): 1.0015 (0.9974, 1.0055)], males [(OR (95% CI): 1.0013 (0.9978, 1.0048)] and females [(OR (95% CI): 1.0016 (0.9973, 1.0059)]. However, the IVW method indicated that there was causal relationship between SUA and rectal cancer risk. The elevated SUA levels could significantly reduce the risk of rectal cancer (OR: 0.9984; 95% CI: 0.9971, 0.9998). The similar result was also found in males (OR: 0.9987; 95% CI: 0.9975, 0.9998) and females (OR: 0.9985; 95% CI: 0.9971, 0.9999) (**Table 2**).

**Table 2:** Mendelian randomization estimates of the causal relationships between serum uric acid levels and cancer risk using inverse variance weighted method.

Population	nsnp	Colorectal cancer		Colon cancer		Rectal Cancer	
		OR (95% CI)	P	OR (95% CI)	P	OR (95% CI)	P
Overall	26	1.0015 (0.9975, 1.0056)	0.459	1.0015 (0.9974, 1.0055)	0.459	0.9984 (0.9971, 0.9998)	0.028
Male	26	0.9989 (0.9968, 1.0009)	0.285	1.0013 (0.9978, 1.0048)	0.459	0.9987 (0.9975, 0.9998)	0.025
Female	26	0.9992 (0.9976, 1.0009)	0.364	1.0016 (0.9973, 1.0059)	0.467	0.9985 (0.9971, 0.9999)	0.037

OR, odds ratio; CI, confidence interval.

### Sensitivity analysis

**Figure 3:**
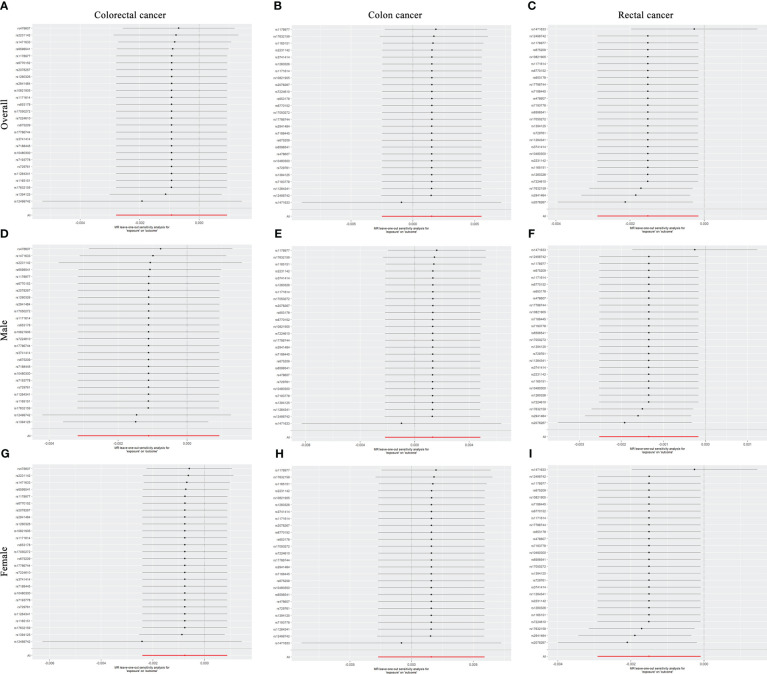
Leave-one-out analyses for serum uric acid levels on cancer.

The Cochran’s Q tests showed that there was no heterogeneity across SNPs. The P values of the MR-Egger regressions were all greater than 0.05, suggesting that there was no horizontal pleiotropy (**Table 3**). The leave-one-out analyses found that rs1471633 in the PDZK1 gene had great possibility to drive the causal association between SUA and rectal cancer risk in overall population, males and females (**Figures 3A–H**). The causal association became not statistically significant if rs1471633 was excluded in the MR analysis (**Figures 3C, F, I**). The MR-Egger regression and weighted median method both showed that there were no causal relationships between SUA and the risk of colorectal cancer, colon cancer and rectal cancer (**Table 4**).

**Table 3:** Heterogeneity and MR-Egger pleiotropy test for the causal relationships between serum uric acid levels and cancer risk.

Phenotype	Cochran’s Q test	P	MR-Egger	SE	P
	Q		Intercept		
Overall					
Colorectal cancer	12.64	0.981	-1.09E04	1.82E-04	0.554
Colon cancer	7.31	0.999	-1.68E04	5.39E-04	0.758
Rectal cancer	9.15	0.998	1.55E-04	1.88E-04	0.420
Male					
Colorectal cancer	12.46	0.982	-9.24E05	2.04E04	0.655
Colon cancer	7.31	0.999	-1.56E04	5.22E04	0.767
Rectal cancer	8.96	0.998	1.51E04	1.72E04	0.389
Female					
Colorectal cancer	12.78	0.979	-1.17E04	1.70E04	0.496
Colon cancer	7.33	0.999	-1.58E04	5.76E04	0.786
Rectal cancer	9.60	0.998	7.62E05	1.99E04	0.705

SE, standard error.

**Table 4:** Mendelian randomization estimates of the causal relationships between serum uric acid levels and cancer risk using MR-Egger and Weighted median method.

Population	nsnp	Colorectal cancer		Colon cancer		Rectal cancer	
		OR (95% CI)	P	OR (95% CI)	P	OR (95% CI)	P
MR-Egger							
Overall	26	1.0046 (0.9851, 1.0245)	0.652	1.0046 (0.9851, 1.0246)	0.652	0.9959 (0.9896, 1.0022)	0.216
Male	26	0.9994 (0.9963, 1.0025)	0.721	1.0038 (0.9874, 1.0205)	0.657	0.9965 (0.9915, 1.0015)	0.181
Female	26	0.9997 (0.9976, 1.0018)	0.773	1.0047 (0.9824, 1.0274)	0.687	0.9972 (0.9903, 1.0041)	0.433
Weighted median							
Overall	26	1.0022 (0.9979, 1.0065)	0.316	1.0022 (0.9979, 1.0065)	0.316	0.9992 (0.9973, 1.0010)	0.357
Male	26	0.9990 (0.9967, 1.0014)	0.433	1.0020 (0.9982, 1.0057)	0.301	0.9993 (0.9977, 1.0008)	0.340
Female	26	0.9937 (0.9977, 1.0011)	0.467	1.0023 (0.9976, 1.0070)	0.339	0.9992 (0.9974, 1.0012)	0.402

OR, odds ratio; CI, confidence interval.

## Discussion

The uric acid is a very important metabolite in human body. It is the end product of the oxidation of xanthine and hypoxanthine catalyzed by xanthine oxidoreductase. This study conducted a two-sample MR study to explore the potential causal relationship between SUA and colorectal cancer risk with GWAS databases available online. The SUA was found causal effect on the risk of rectal cancer with IVW method, but the sensitivity analyses did not support the finding. The similar result was also observed in males and females, respectively.

More and more studies supported the carcinogenic effect of SUA. It was reported that the activation of xanthine oxidase during SUA production leads to the increase of reactive oxygen species (ROS) *in vivo* (21). ROS activated signaling pathways related to proliferation, survival, angiogenesis and metastasis, promote the occurrence, development and metastasis of tumors (22, 23). SUA could also cause the upregulation of mRNA levels of inflammasome‐related genes (e.g. IL‐1β and NLRP3) through monosodium urate crystals and soluble uric acid, giving rise to oncogenes potential (24, 25). Yiu et al. conducted a cohort study on 493,281 persons and found that altered SUA levels were associated with the risk of hepatobiliary, kidney, nonmelanoma skin and other cancers in males, head and neck and other cancers in females (12). An UK cohort study including 444,462 participants showed that high SUA levels were associated with an increased risk of pancreatic cancer and kidney cancer in females and gallbladder cancer in males (26, 27). SUA was also found positively associated with the risk of prostate, esophagus, stomach, liver, pancreatic, lung, ovarian, renal and bladder cancers in two prospective studies in Korean population (28, 29). A meta-analysis found that gout was a risk factor of urological cancers, digestive system cancers and lung cancer (30). Of the above reported cancers that were positively associated with SUA, only non-tobacco related cancer and prostate cancer were found causal relationships with SUA in published MR studies (16, 17). Similarly, several cohort studies reported that high SUA levels increased the risk of colorectal cancer in Swedish (12) and cancer in Chinese males (13). However, this MR study did not find positive causal associations between SUA and the risk of colorectal cancer, colon cancer and rectal cancer. It implied that the results of previous cohort studies may be affected by unobservable confounding factors.

Ames et al. firstly proposed that SUA as an antioxidant and scavenger of free radicals was working on singlet oxygen which can reduce the risk of various cancer types (31). The urate could activate potent immune stimulants and trigger anticancer immune response by reversing immunosuppression or as an adjuvant (32). In this MR study, it was found that SUA was a protective agent against rectal cancer and the protective effect of SUA on rectal cancer risk was influenced by rs1471633. The rs1471633 was reported to correlate with the decreasing trend of PDZK1 mRNA, whose protein had been shown to be a key regulatory scaffolding protein in tethering urate transportsome complex (33, 34). The previous studies reported inconsistent expression levels of PDZK1 across different cancers. The low-level PDZK1 expression had been reported in renal cell carcinoma (35) and gastric cancer (36), and exhibited tumor suppressive effects. In contrast, the PDZK1 expression was upregulated in breast cancer (37), thyroid cancer (38) and hepatocellular carcinoma (39). Therefore, further research is needed to explore the effect of PDZK1 expression on rectal cancer risk. However, the causal association between SUA and rectal cancer risk was not found in the MR-Egger regression and weighted median method. Given the robustness of results, the SUA levels were considered no causal relationship with rectal cancer risk.

Mi et al. reported that lowest uric acid groups (≤3.5 mg/dL) and highest uric acid groups (>8.4 mg/dL) both had higher risk of colorectal cancer compared with the reference groups (4.4~5.4 mg/dl), and concluded the U-shaped association between SUA and colorectal cancer risk (14). It was demonstrated that SUA was a powerful antioxidant at physiological concentrations, while it was a pro-oxidant molecule at high intracellular concentrations (40). The SUA levels as continuous variable in this MR study were relatively in the normal range which may limit the correlation analyses between SUA and colorectal cancer risk. Besides, the results of correlation analyses may vary when SUA had different types of variables in the models. Therefore, further research is needed to investigate the role of higher SUA levels such as hyperuricemia or gout in the occurrence of colorectal cancer.

This study had some strengths. First, MR analysis was used to analyze the causal effects of SUA on colorectal cancer risk to avoid confounding and reverse causality biases. The sensitivity analyses were applied to increase the robustness of the obtained results. Second, the data of colon cancer and rectal cancer from more variety of databases were abstracted to assess the causal effects of SUA on colorectal cancer risk. Moreover, meta-analyses with random effect models incorporated all available GWASs summary data to analyze the genetic association with colorectal cancer.

Several issues should be considered in this study. First, the causal effect obtained in MR study did not indicate an effect on the risk of colorectal cancer after changing SUA levels. Second, the causality between SUA and colorectal cancer risk may also vary by within or out of the normal range for SUA levels. The SUA levels may not be a simple linear association but a U-shaped or J-shaped association with cancer risk. This study was limited to the absence of information about SNPs-SUA and SNPs-cancer stratified by SUA levels in the available GWAS datasets. Third, the results may only be extrapolated to the European population. Future studies could consider other ethnicities and appropriate stratification analyses.

## Conclusions

Overall, there was no linear causal association between SUA and the risk of colorectal cancer. However, further research is needed to investigate the role of higher SUA levels such as hyperuricemia or gout in the occurrence of colorectal cancer.

## Data availability statement

Publicly available datasets were analyzed in this study. This data can be found here: The GWASs used in this study can be available from the latest MRC Integrative Epidemiology Unit GWAS database at https://gwas.mrcieu.ac.uk.

## Author contributions

JSZ: Formal Analysis, Writing – original draft, Writing – review & editing. RF: Conceptualization, Writing – original draft, Writing – review & editing. JWZ: Validation, Writing – original draft. SHZ: Validation, Writing – original draft. ZFL: Validation, Writing – original draft. ZL: Project administration, Writing – review & editing. XL: Data curation, Writing – original draft. XLX: Data curation, Writing – original draft. YLC: Data curation, Writing – original draft. ZJH: Conceptualization, Supervision, Writing – review & editing.
