# Supplementary material for: Association between serum uric acid and colorectal cancer risk in European population: a two-sample Mendelian randomization study
Source: Front Oncol. 2024 Jul 1;14:1394320. doi: 10.3389/fonc.2024.1394320 (PMC11246881; doi:10.3389/fonc.2024.1394320)
Supplement: Supplementary file 1 [file DataSheet_1.pdf]

Supplementary Tables

Supplementary Table S1 Characteristics of 26 serum uric acid-associated SNPs

SNP	Chr	BP (Build 36)	Gene	EA	OA	EAF	β	SE	P	F-statistic
rs1471633	1	144435096	PDZK1	A	C	0.46	0.059	0.005	1.2E-29	139.06
rs11264341	1	153418117	TRIM46	T	C	0.43	-0.05	0.006	6.2E-19	69.40
rs1260326	2	27584444	GCKR	T	C	0.41	0.074	0.005	1.2E-44	218.60
rs17050272	2	121022910	INHBB	A	G	0.43	0.035	0.006	1.6E-10	34.02
rs6770152	3	53075254	SFMBT1	T	G	0.58	-0.044	0.005	2.6E-16	77.38
rs12498742	4	9553150	SLC2A9	A	G	0.77	0.373	0.006	<1.0E-300	3733.82
rs2231142	4	89271347	ABCG2	T	G	0.11	0.217	0.009	1.0E-134	57.28
rs17632159	5	72467238	TMEM171	C	G	0.31	-0.039	0.006	3.5E-11	42.23
rs675209	6	7047083	RREB1	T	C	0.27	0.061	0.006	1.3E-23	103.26
rs1165151	6	25929595	SLC17A1	T	G	0.47	-0.091	0.005	7.0E-70	330.24
rs729761	6	43912549	VEGFA	T	G	0.30	-0.047	0.006	8.0E-16	61.33
rs1178977	7	72494985	BAZ1B	A	G	0.81	0.047	0.007	1.2E-12	45.06
rs10480300	7	151036938	PRKAG2	T	C	0.28	0.035	0.006	4.1E-09	34.02
rs17786744	8	23832951	STC1	A	G	0.58	-0.029	0.005	1.4E-08	33.63
rs2941484	8	76641323	HNF4G	T	C	0.44	0.044	0.005	4.4E-17	77.38
rs1171614	10	61139544	SLC16A9	T	C	0.22	-0.079	0.007	2.3E-28	127.22
rs10821905	10	52316099	A1CF	A	G	0.18	0.057	0.007	7.4E-17	66.26
rs2078267	11	64090690	SLC22A11	T	C	0.51	-0.073	0.006	9.4E-38	147.83
rs478607	11	64234639	NRXN2	A	G	0.84	-0.047	0.007	4.4E-11	45.06
rs3741414	12	56130316	INHBC	T	C	0.24	-0.072	0.007	2.2E-25	48.98
rs653178	12	110492139	ATXN2	T	C	0.51	-0.035	0.005	7.2E-12	48.98
rs1394125	15	73946038	UBE2Q2	A	G	0.34	0.043	0.006	2.5E-13	51.34
rs6598541	15	97088658	IGF1R	A	G	0.36	0.043	0.006	4.8E-15	51.34
rs7193778	16	68121391	NFAT5	T	C	0.86	-0.046	0.008	8.2E-10	30.05
rs7188445	16	78292488	MAF	A	G	0.33	-0.032	0.005	1.6E-09	40.94
rs7224610	17	50719787	HLF	A	C	0.58	-0.042	0.005	5.4E-17	70.51

SNP: single-nucleotide polymorphism; Chr: chromosome; EA: effect allele; OA: other allele; EAF: effect allelea frequency; β: regression coefficient; SE: standard error.

Supplementary Table S2 Gender-specific effects for 26 serum uric acid-associated SNPs

SNP	Chr	EA	OA	Males-specific effect			Females-specific effect		
				β	SE	P	β	SE	P
rs1471633	1	A	C	0.069	0.008	3.5E-15	0.054	0.007	1.6E-14
rs11264341	1	T	C	-0.055	0.009	1.1E-08	-0.044	0.007	9.1E-09
rs1260326	2	T	C	0.091	0.009	3.0E-25	0.063	0.007	1.1E-18
rs17050272	2	A	G	0.049	0.010	6.5E-07	0.030	0.008	1.9E-04
rs6770152	3	T	G	-0.052	0.009	6.7E-09	-0.047	0.007	6.0E-11
rs12498742	4	A	G	0.269	0.010	6.4E-153	0.460	0.008	<1.0E-700
rs2231142	4	T	G	0.270	0.014	3.8E-75	0.181	0.011	1.3E-52
rs17632159	5	C	G	-0.043	0.010	1.3E-05	-0.039	0.008	1.1E-06
rs675209	6	T	C	0.060	0.010	3.3E-09	0.064	0.008	2.0E-15
rs1165151	6	T	G	-0.096	0.008	1.3E-28	-0.089	0.007	4.2E-37
rs729761	6	T	G	-0.047	0.010	3.2E-06	-0.047	0.008	8.1E-09
rs1178977	7	A	G	0.055	0.011	8.2E-07	0.046	0.009	2.6E-07
rs10480300	7	T	C	0.043	0.010	1.7E-05	0.024	0.008	3.2E-03
rs17786744	8	A	G	-0.033	0.009	2.1E-04	-0.029	0.007	5.0E-05
rs2941484	8	T	C	0.048	0.009	6.2E-08	0.046	0.007	1.3E-10
rs1171614	10	T	C	-0.086	0.011	1.9E-13	-0.067	0.009	3.0E-13
rs10821905	10	A	G	0.042	0.011	3.8E-04	0.060	0.009	2.5E-10
rs2078267	11	T	C	-0.085	0.009	2.9E-19	-0.071	0.007	5.7E-20
rs478607	11	A	G	-0.058	0.012	9.6E-07	-0.043	0.009	8.8E-06
rs3741414	12	T	C	-0.091	0.011	7.0E-16	-0.057	0.009	4.3E-10
rs653178	12	T	C	-0.085	0.009	2.9E-19	-0.071	0.007	5.7E-20
rs1394125	15	A	G	0.060	0.010	6.3E-09	0.032	0.008	1.0E-04
rs6598541	15	A	G	0.039	0.009	2.7E-05	0.050	0.007	1.6E-11
rs7193778	16	T	C	-0.048	0.012	2.1E-04	-0.045	0.010	1.0E-05
rs7188445	16	A	G	-0.025	0.009	7.9E-03	-0.040	0.007	6.4E-08
rs7224610	17	A	C	-0.043	0.009	9.0E-07	-0.034	0.007	3.0E-06

SNP: single-nucleotide polymorphism; Chr: chromosome; EA: effect allele; OA: other allele; β: regression coefficient; SE: standard error.

Supplementary Figures

Supplementary Figure S1 Serum uric acid levels in 48 studies used to explore serum uric-associated SNPs.

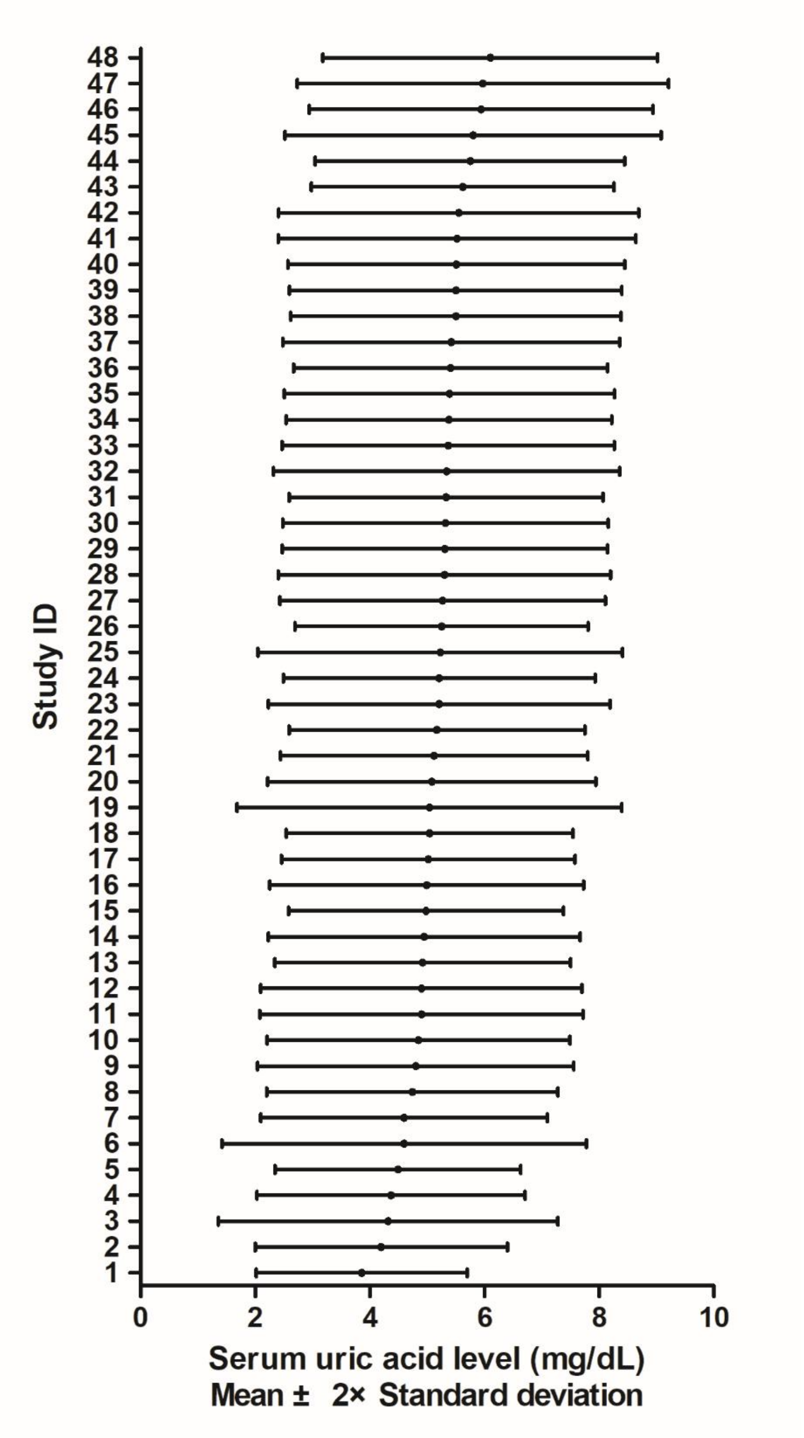

Supplementary Figure S2 Forest plot of the correlation between SNPs and colorectal cancer.
GWAS: genome-wide association study; OR: odds ratio; CI: confidence interval.

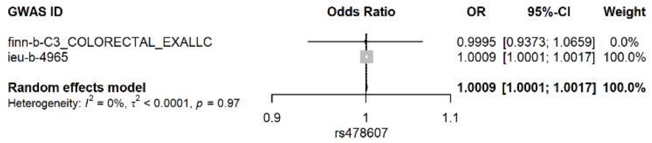

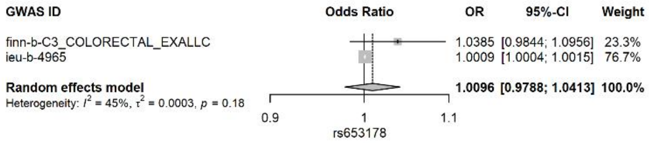

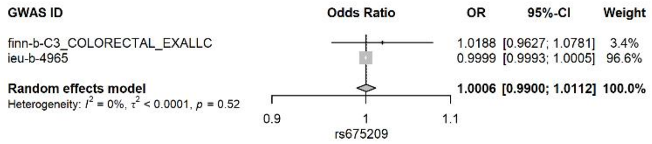

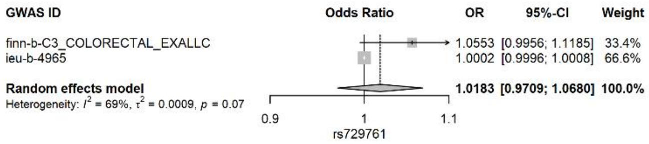

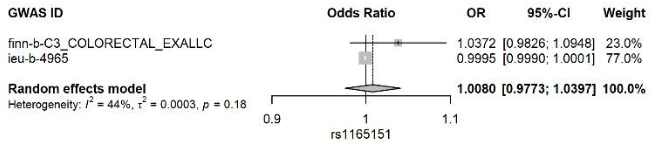

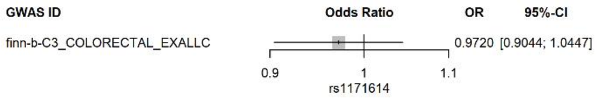

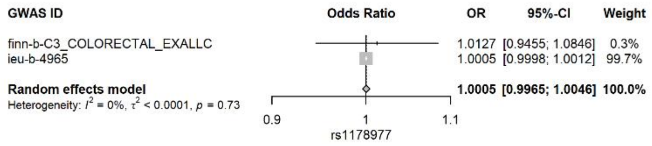

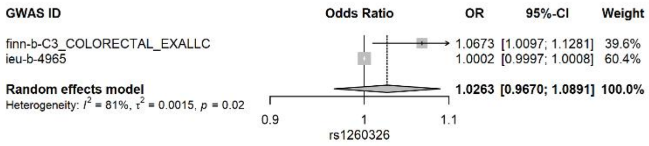

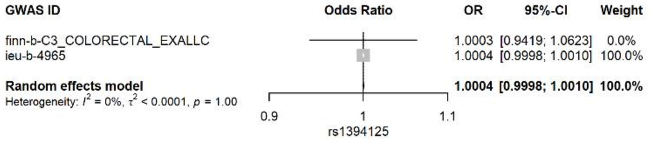
GWAS ID
Odds Ratio
OR
95%-CI
Weight
finn-b-C3_COLORECTAL_EXALLC 1.0003 [0.9419; 1.0623] 0.0%
ieu-b-4965 1.0004 [0.9998; 1.0010] 100.0%
Random effects model 1.0004 [0.9998; 1.0010] 100.0%
Heterogeneity: I^2 = 0%, τ^2 < 0.0001, p = 1.00
0.9
1
1.1
rs1394125

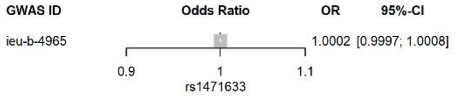
GWAS ID
Odds Ratio
OR
95%-CI
ieu-b-4965 1.0002 [0.9997; 1.0008]
0.9
1
1.1
rs1471633

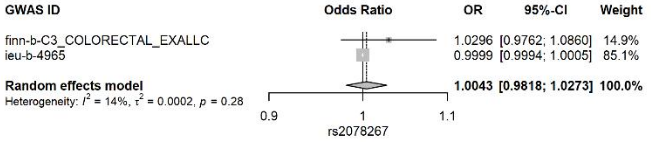
GWAS ID
Odds Ratio
OR
95%-CI
Weight
finn-b-C3_COLORECTAL_EXALLC 1.0296 [0.9762; 1.0860] 14.9%
ieu-b-4965 0.9999 [0.9994; 1.0005] 85.1%
Random effects model 1.0043 [0.9818; 1.0273] 100.0%
Heterogeneity: I^2 = 14%, τ^2 = 0.0002, p = 0.28
0.9
1
1.1
rs2078267

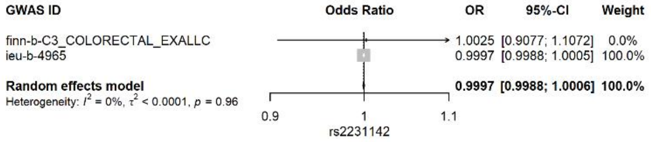
GWAS ID
Odds Ratio
OR
95%-CI
Weight
finn-b-C3_COLORECTAL_EXALLC 1.0025 [0.9077; 1.1072] 0.0%
ieu-b-4965 0.9997 [0.9988; 1.0005] 100.0%
Random effects model 0.9997 [0.9988; 1.0006] 100.0%
Heterogeneity: I^2 = 0%, τ^2 < 0.0001, p = 0.96
0.9
1
1.1
rs2231142

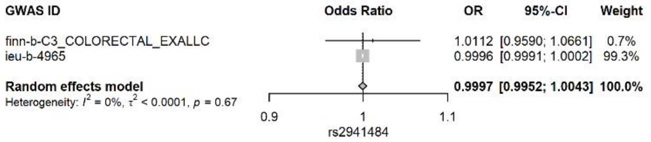
GWAS ID
Odds Ratio
OR
95%-CI
Weight
finn-b-C3_COLORECTAL_EXALLC 1.0112 [0.9590; 1.0661] 0.7%
ieu-b-4965 0.9996 [0.9991; 1.0002] 99.3%
Random effects model 0.9997 [0.9952; 1.0043] 100.0%
Heterogeneity: I^2 = 0%, τ^2 < 0.0001, p = 0.67
0.9
1
1.1
rs2941484

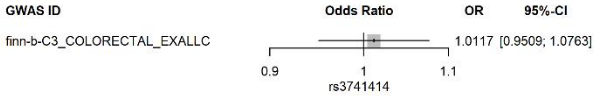
GWAS ID
Odds Ratio
OR
95%-CI
finn-b-C3_COLORECTAL_EXALLC 1.0117 [0.9509; 1.0763]
0.9
1
1.1
rs3741414

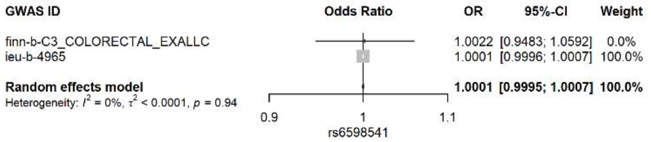
GWAS ID
Odds Ratio
OR
95%-CI
Weight
finn-b-C3_COLORECTAL_EXALLC 1.0022 [0.9483; 1.0592] 0.0%
ieu-b-4965 1.0001 [0.9996; 1.0007] 100.0%
Random effects model 1.0001 [0.9995; 1.0007] 100.0%
Heterogeneity: I^2 = 0%, τ^2 < 0.0001, p = 0.94
0.9
1
1.1
rs6598541

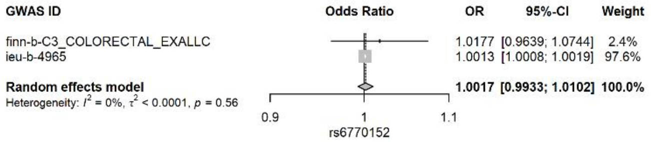
GWAS ID
Odds Ratio
OR
95%-CI
Weight
finn-b-C3_COLORECTAL_EXALLC 1.0177 [0.9639; 1.0744] 2.4%
ieu-b-4965 1.0013 [1.0008; 1.0019] 97.6%
Random effects model 1.0017 [0.9933; 1.0102] 100.0%
Heterogeneity: I^2 = 0%, τ^2 < 0.0001, p = 0.56
0.9
1
1.1
rs6770152

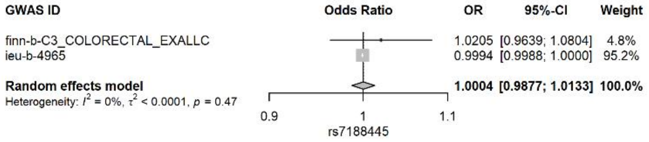
GWAS ID
Odds Ratio
OR
95%-CI
Weight
finn-b-C3_COLORECTAL_EXALLC
ieu-b-4965
1.0205 [0.9639; 1.0804] 4.8%
0.9994 [0.9988; 1.0000] 95.2%
Random effects model
Heterogeneity: I^2 = 0%, τ^2 < 0.0001, p = 0.47
1.0004 [0.9877; 1.0133] 100.0%
0.9
1
1.1
rs7188445

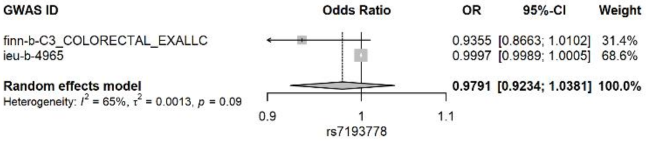
GWAS ID
Odds Ratio
OR
95%-CI
Weight
finn-b-C3_COLORECTAL_EXALLC
ieu-b-4965
0.9355 [0.8663; 1.0102] 31.4%
0.9997 [0.9989; 1.0005] 68.6%
Random effects model
Heterogeneity: I^2 = 65%, τ^2 = 0.0013, p = 0.09
0.9791 [0.9234; 1.0381] 100.0%
0.9
1
1.1
rs7193778

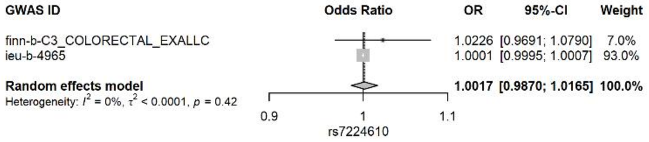
GWAS ID
Odds Ratio
OR
95%-CI
Weight
finn-b-C3_COLORECTAL_EXALLC
ieu-b-4965
1.0226 [0.9691; 1.0790] 7.0%
1.0001 [0.9995; 1.0007] 93.0%
Random effects model
Heterogeneity: I^2 = 0%, τ^2 < 0.0001, p = 0.42
1.0017 [0.9870; 1.0165] 100.0%
0.9
1
1.1
rs7224610

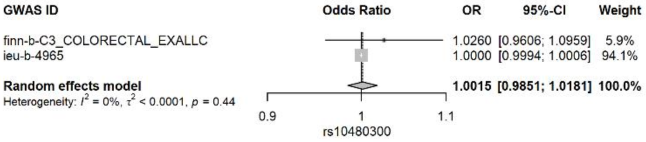
GWAS ID
Odds Ratio
OR
95%-CI
Weight
finn-b-C3_COLORECTAL_EXALLC
ieu-b-4965
1.0260 [0.9606; 1.0959] 5.9%
1.0000 [0.9994; 1.0006] 94.1%
Random effects model
Heterogeneity: I^2 = 0%, τ^2 < 0.0001, p = 0.44
1.0015 [0.9851; 1.0181] 100.0%
0.9
1
1.1
rs10480300

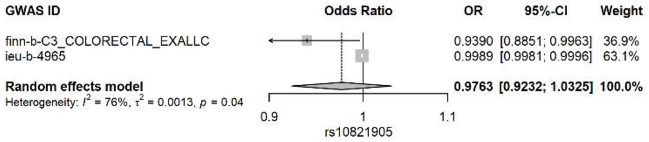
GWAS ID
Odds Ratio
OR
95%-CI
Weight
finn-b-C3_COLORECTAL_EXALLC
ieu-b-4965
0.9390 [0.8851; 0.9963] 36.9%
0.9989 [0.9981; 0.9996] 63.1%
Random effects model
Heterogeneity: I^2 = 76%, τ^2 = 0.0013, p = 0.04
0.9763 [0.9232; 1.0325] 100.0%
0.9
1
1.1
rs10821905

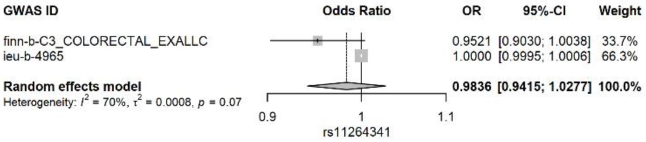
GWAS ID
Odds Ratio
OR
95%-CI
Weight
finn-b-C3_COLORECTAL_EXALLC
ieu-b-4965
0.9521 [0.9030; 1.0038] 33.7%
1.0000 [0.9995; 1.0006] 66.3%
Random effects model
Heterogeneity: I^2 = 70%, τ^2 = 0.0008, p = 0.07
0.9836 [0.9415; 1.0277] 100.0%
0.9
1
1.1
rs11264341

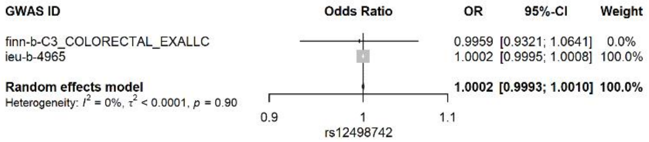
GWAS ID
Odds Ratio
OR
95%-CI
Weight
finn-b-C3_COLORECTAL_EXALLC
ieu-b-4965
0.9959 [0.9321; 1.0641] 0.0%
1.0002 [0.9995; 1.0008] 100.0%
Random effects model
Heterogeneity: I^2 = 0%, τ^2 < 0.0001, p = 0.90
1.0002 [0.9993; 1.0010] 100.0%
0.9
1
1.1
rs12498742

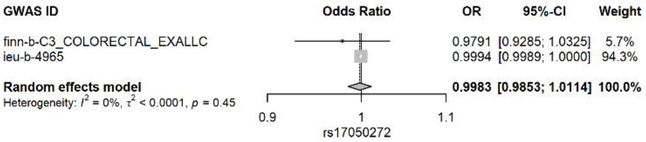
GWAS ID
Odds Ratio
OR
95%-CI
Weight
finn-b-C3_COLORECTAL_EXALLC
ieu-b-4965
0.9791 [0.9285; 1.0325] 5.7%
0.9994 [0.9989; 1.0000] 94.3%
Random effects model
Heterogeneity: I^2 = 0%, τ^2 < 0.0001, p = 0.45
0.9983 [0.9853; 1.0114] 100.0%
0.9
1
1.1
rs17050272

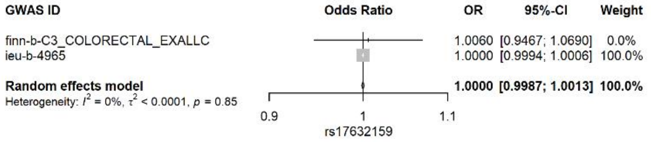
GWAS ID
Odds Ratio
OR
95%-CI
Weight
finn-b-C3_COLORECTAL_EXALLC
1.0060 [0.9467; 1.0690] 0.0%
ieu-b-4965
1.0000 [0.9994; 1.0006] 100.0%
Random effects model
1.0000 [0.9987; 1.0013] 100.0%
Heterogeneity: I^2 = 0%, τ^2 < 0.0001, p = 0.85
0.9
1
1.1
rs17632159

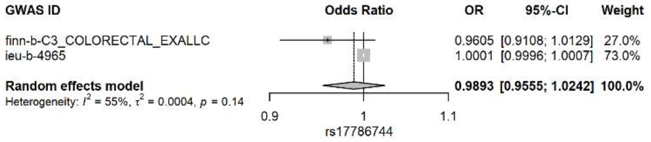
GWAS ID
Odds Ratio
OR
95%-CI
Weight
finn-b-C3_COLORECTAL_EXALLC
0.9605 [0.9108; 1.0129] 27.0%
ieu-b-4965
1.0001 [0.9996; 1.0007] 73.0%
Random effects model
0.9893 [0.9555; 1.0242] 100.0%
Heterogeneity: I^2 = 55%, τ^2 = 0.0004, p = 0.14
0.9
1
1.1
rs17786744

Supplementary Figure S3 Forest plot of the correlation between SNPs and colon cancer.

GWAS: genome-wide association study; OR: odds ratio; CI: confidence interval.

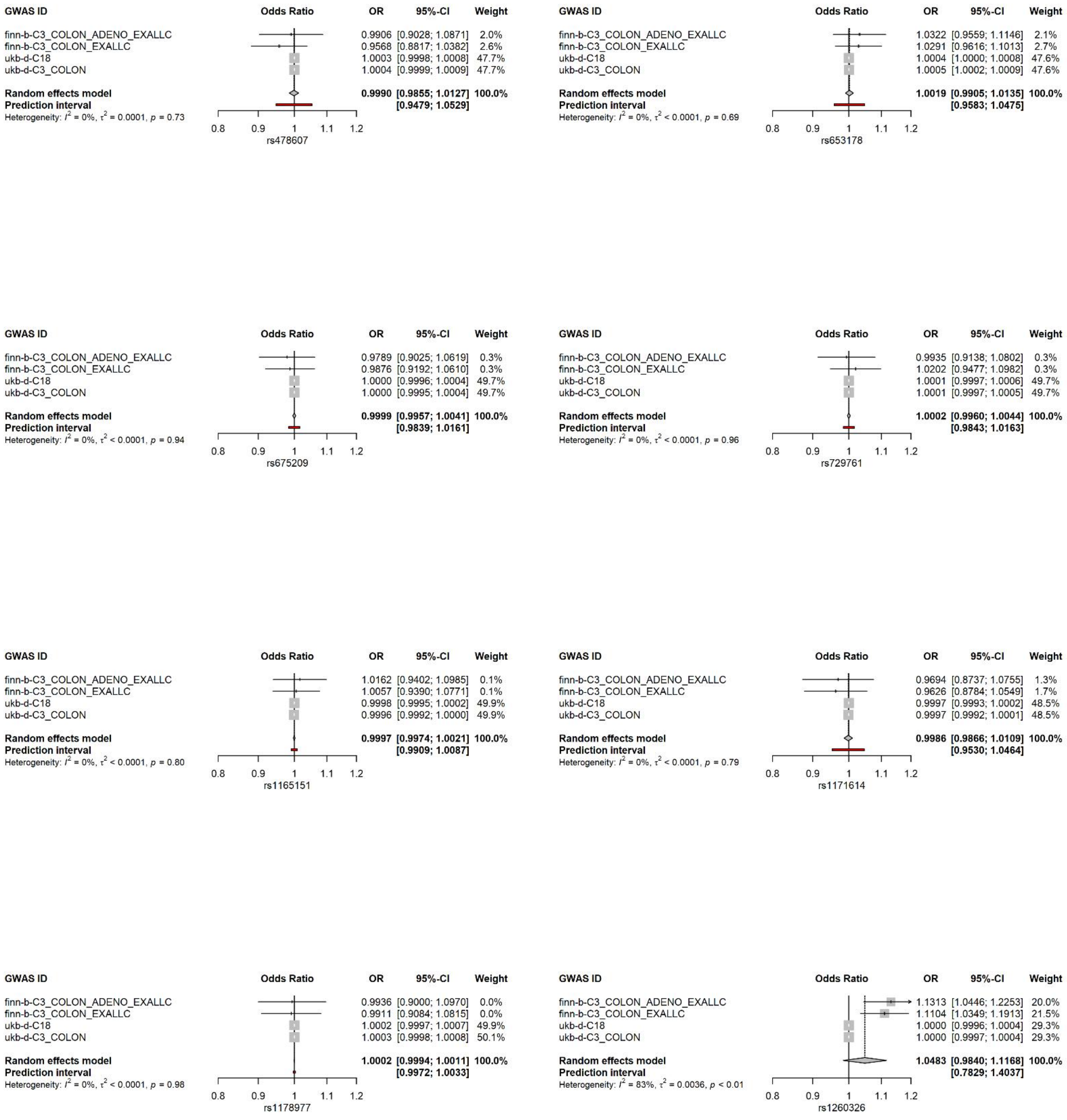

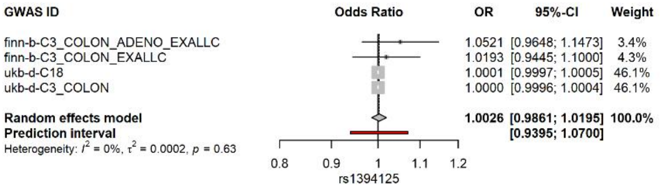
GWAS ID
Odds Ratio
OR
95%-CI
Weight
finn-b-C3_COLON_ADENO_EXALLC 1.0521 [0.9648; 1.1473] 3.4%
finn-b-C3_COLON_EXALLC 1.0193 [0.9445; 1.1000] 4.3%
ukb-d-C18 1.0001 [0.9997; 1.0005] 46.1%
ukb-d-C3_COLON 1.0000 [0.9996; 1.0004] 46.1%
Random effects model 1.0026 [0.9861; 1.0195] 100.0%
Prediction interval [0.9395; 1.0700]
Heterogeneity: $I^2 = 0\%$, $\tau^2 = 0.0002$, $p = 0.63$
0.8 0.9 1 1.1 1.2
rs1394125

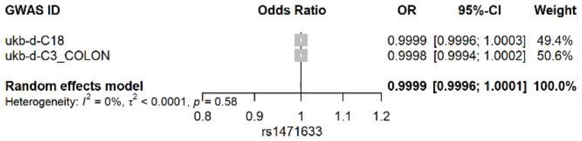
GWAS ID
Odds Ratio
OR
95%-CI
Weight
ukb-d-C18 0.9999 [0.9996; 1.0003] 49.4%
ukb-d-C3_COLON 0.9998 [0.9994; 1.0002] 50.6%
Random effects model 0.9999 [0.9996; 1.0001] 100.0%
Heterogeneity: $I^2 = 0\%$, $\tau^2 < 0.0001$, $p = 0.58$
0.8 0.9 1 1.1 1.2
rs1471633

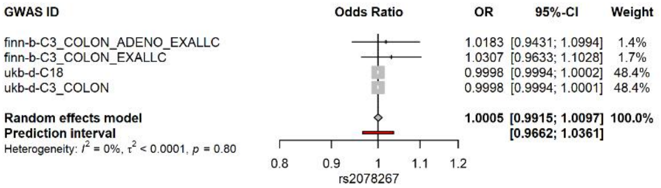
GWAS ID
Odds Ratio
OR
95%-CI
Weight
finn-b-C3_COLON_ADENO_EXALLC 1.0183 [0.9431; 1.0994] 1.4%
finn-b-C3_COLON_EXALLC 1.0307 [0.9633; 1.1028] 1.7%
ukb-d-C18 0.9998 [0.9994; 1.0002] 48.4%
ukb-d-C3_COLON 0.9998 [0.9994; 1.0001] 48.4%
Random effects model 1.0005 [0.9915; 1.0097] 100.0%
Prediction interval [0.9662; 1.0361]
Heterogeneity: $I^2 = 0\%$, $\tau^2 < 0.0001$, $p = 0.80$
0.8 0.9 1 1.1 1.2
rs2078267

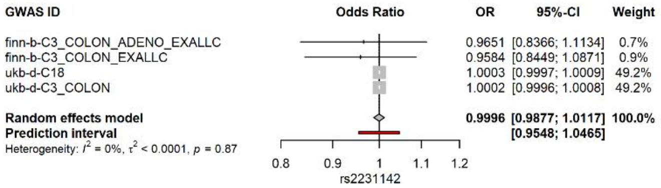
GWAS ID
Odds Ratio
OR
95%-CI
Weight
finn-b-C3_COLON_ADENO_EXALLC 0.9651 [0.8366; 1.1134] 0.7%
finn-b-C3_COLON_EXALLC 0.9584 [0.8449; 1.0871] 0.9%
ukb-d-C18 1.0003 [0.9997; 1.0009] 49.2%
ukb-d-C3_COLON 1.0002 [0.9996; 1.0008] 49.2%
Random effects model 0.9996 [0.9877; 1.0117] 100.0%
Prediction interval [0.9548; 1.0465]
Heterogeneity: $I^2 = 0\%$, $\tau^2 < 0.0001$, $p = 0.87$
0.8 0.9 1 1.1 1.2
rs2231142

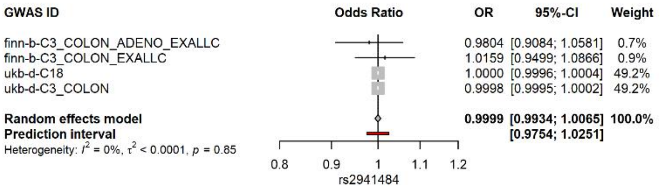
GWAS ID
Odds Ratio
OR
95%-CI
Weight
finn-b-C3_COLON_ADENO_EXALLC 0.9804 [0.9084; 1.0581] 0.7%
finn-b-C3_COLON_EXALLC 1.0159 [0.9499; 1.0866] 0.9%
ukb-d-C18 1.0000 [0.9996; 1.0004] 49.2%
ukb-d-C3_COLON 0.9998 [0.9995; 1.0002] 49.2%
Random effects model 0.9999 [0.9934; 1.0065] 100.0%
Prediction interval [0.9754; 1.0251]
Heterogeneity: $I^2 = 0\%$, $\tau^2 < 0.0001$, $p = 0.85$
0.8 0.9 1 1.1 1.2
rs2941484

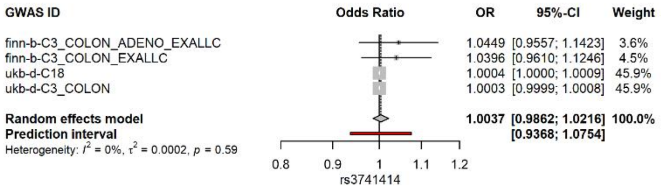
GWAS ID
Odds Ratio
OR
95%-CI
Weight
finn-b-C3_COLON_ADENO_EXALLC 1.0449 [0.9557; 1.1423] 3.6%
finn-b-C3_COLON_EXALLC 1.0396 [0.9610; 1.1246] 4.5%
ukb-d-C18 1.0004 [1.0000; 1.0009] 45.9%
ukb-d-C3_COLON 1.0003 [0.9999; 1.0008] 45.9%
Random effects model 1.0037 [0.9862; 1.0216] 100.0%
Prediction interval [0.9368; 1.0754]
Heterogeneity: $I^2 = 0\%$, $\tau^2 = 0.0002$, $p = 0.59$
0.8 0.9 1 1.1 1.2
rs3741414

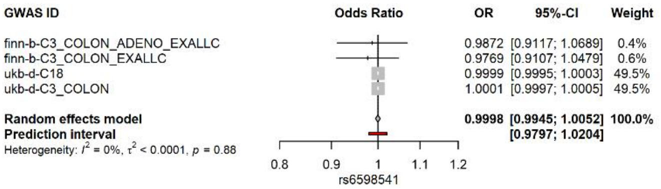
GWAS ID
Odds Ratio
OR
95%-CI
Weight
finn-b-C3_COLON_ADENO_EXALLC 0.9872 [0.9117; 1.0689] 0.4%
finn-b-C3_COLON_EXALLC 0.9769 [0.9107; 1.0479] 0.6%
ukb-d-C18 0.9999 [0.9995; 1.0003] 49.5%
ukb-d-C3_COLON 1.0001 [0.9997; 1.0005] 49.5%
Random effects model 0.9998 [0.9945; 1.0052] 100.0%
Prediction interval [0.9797; 1.0204]
Heterogeneity: $I^2 = 0\%$, $\tau^2 < 0.0001$, $p = 0.88$
0.8 0.9 1 1.1 1.2
rs6598541

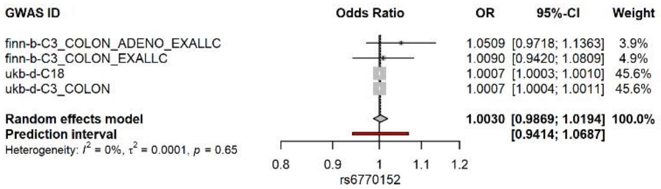
GWAS ID
Odds Ratio
OR
95%-CI
Weight
finn-b-C3_COLON_ADENO_EXALLC 1.0509 [0.9718; 1.1363] 3.9%
finn-b-C3_COLON_EXALLC 1.0090 [0.9420; 1.0809] 4.9%
ukb-d-C18 1.0007 [1.0003; 1.0010] 45.6%
ukb-d-C3_COLON 1.0007 [1.0004; 1.0011] 45.6%
Random effects model 1.0030 [0.9869; 1.0194] 100.0%
Prediction interval [0.9414; 1.0687]
Heterogeneity: $I^2 = 0\%$, $\tau^2 = 0.0001$, $p = 0.65$
0.8 0.9 1 1.1 1.2
rs6770152

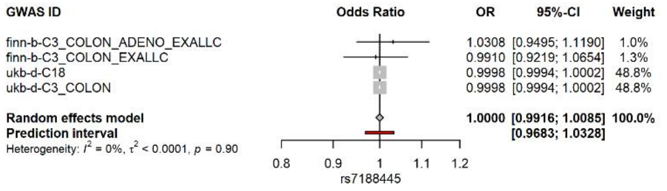
GWAS ID
Odds Ratio
OR
95%-CI
Weight
finn-b-C3_COLON_ADENO_EXALLC 1.0308 [0.9495; 1.1190] 1.0%
finn-b-C3_COLON_EXALLC 0.9910 [0.9219; 1.0654] 1.3%
ukb-d-C18 0.9998 [0.9994; 1.0002] 48.8%
ukb-d-C3_COLON 0.9998 [0.9994; 1.0002] 48.8%
Random effects model 1.0000 [0.9916; 1.0085] 100.0%
Prediction interval [0.9683; 1.0328]
Heterogeneity: I^2 = 0%, τ^2 < 0.0001, p = 0.90
0.8 0.9 1 1.1 1.2
rs7188445

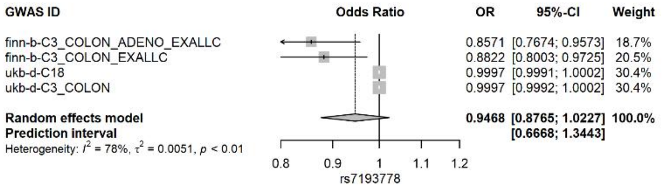
GWAS ID
Odds Ratio
OR
95%-CI
Weight
finn-b-C3_COLON_ADENO_EXALLC 0.8571 [0.7674; 0.9573] 18.7%
finn-b-C3_COLON_EXALLC 0.8822 [0.8003; 0.9725] 20.5%
ukb-d-C18 0.9997 [0.9991; 1.0002] 30.4%
ukb-d-C3_COLON 0.9997 [0.9992; 1.0002] 30.4%
Random effects model 0.9468 [0.8765; 1.0227] 100.0%
Prediction interval [0.6668; 1.3443]
Heterogeneity: I^2 = 78%, τ^2 = 0.0051, p < 0.01
0.8 0.9 1 1.1 1.2
rs7193778

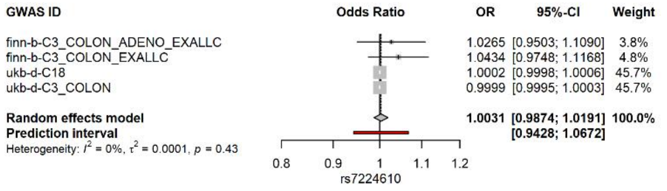
GWAS ID
Odds Ratio
OR
95%-CI
Weight
finn-b-C3_COLON_ADENO_EXALLC 1.0265 [0.9503; 1.1090] 3.8%
finn-b-C3_COLON_EXALLC 1.0434 [0.9748; 1.1168] 4.8%
ukb-d-C18 1.0002 [0.9998; 1.0006] 45.7%
ukb-d-C3_COLON 0.9999 [0.9995; 1.0003] 45.7%
Random effects model 1.0031 [0.9874; 1.0191] 100.0%
Prediction interval [0.9428; 1.0672]
Heterogeneity: I^2 = 0%, τ^2 = 0.0001, p = 0.43
0.8 0.9 1 1.1 1.2
rs7224610

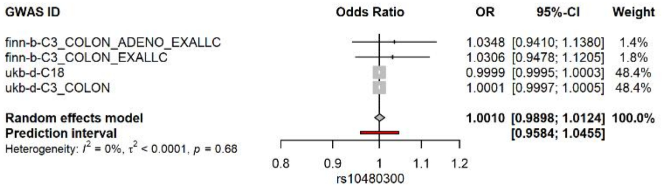
GWAS ID
Odds Ratio
OR
95%-CI
Weight
finn-b-C3_COLON_ADENO_EXALLC 1.0348 [0.9410; 1.1380] 1.4%
finn-b-C3_COLON_EXALLC 1.0306 [0.9478; 1.1205] 1.8%
ukb-d-C18 0.9999 [0.9995; 1.0003] 48.4%
ukb-d-C3_COLON 1.0001 [0.9997; 1.0005] 48.4%
Random effects model 1.0010 [0.9898; 1.0124] 100.0%
Prediction interval [0.9584; 1.0455]
Heterogeneity: I^2 = 0%, τ^2 < 0.0001, p = 0.68
0.8 0.9 1 1.1 1.2
rs10480300

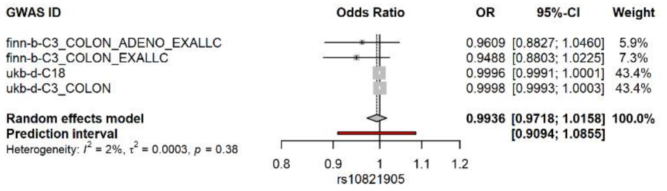
GWAS ID
Odds Ratio
OR
95%-CI
Weight
finn-b-C3_COLON_ADENO_EXALLC 0.9609 [0.8827; 1.0460] 5.9%
finn-b-C3_COLON_EXALLC 0.9488 [0.8803; 1.0225] 7.3%
ukb-d-C18 0.9996 [0.9991; 1.0001] 43.4%
ukb-d-C3_COLON 0.9998 [0.9993; 1.0003] 43.4%
Random effects model 0.9936 [0.9718; 1.0158] 100.0%
Prediction interval [0.9094; 1.0855]
Heterogeneity: I^2 = 2%, τ^2 = 0.0003, p = 0.38
0.8 0.9 1 1.1 1.2
rs10821905

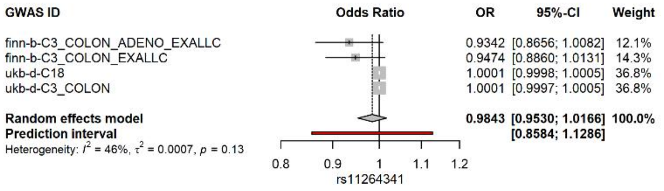
GWAS ID
Odds Ratio
OR
95%-CI
Weight
finn-b-C3_COLON_ADENO_EXALLC 0.9342 [0.8656; 1.0082] 12.1%
finn-b-C3_COLON_EXALLC 0.9474 [0.8860; 1.0131] 14.3%
ukb-d-C18 1.0001 [0.9998; 1.0005] 36.8%
ukb-d-C3_COLON 1.0001 [0.9997; 1.0005] 36.8%
Random effects model 0.9843 [0.9530; 1.0166] 100.0%
Prediction interval [0.8584; 1.1286]
Heterogeneity: I^2 = 46%, τ^2 = 0.0007, p = 0.13
0.8 0.9 1 1.1 1.2
rs11264341

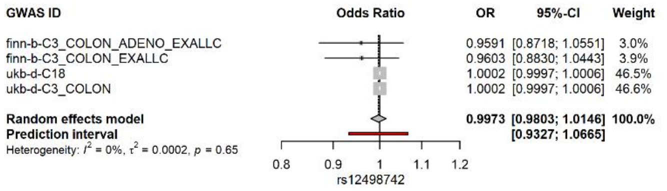
GWAS ID
Odds Ratio
OR
95%-CI
Weight
finn-b-C3_COLON_ADENO_EXALLC 0.9591 [0.8718; 1.0551] 3.0%
finn-b-C3_COLON_EXALLC 0.9603 [0.8830; 1.0443] 3.9%
ukb-d-C18 1.0002 [0.9997; 1.0006] 46.5%
ukb-d-C3_COLON 1.0002 [0.9997; 1.0006] 46.6%
Random effects model 0.9973 [0.9803; 1.0146] 100.0%
Prediction interval [0.9327; 1.0665]
Heterogeneity: I^2 = 0%, τ^2 = 0.0002, p = 0.65
0.8 0.9 1 1.1 1.2
rs12498742

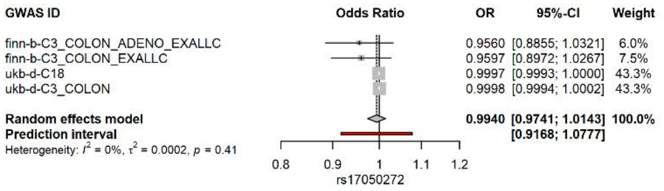
GWAS ID
Odds Ratio
OR
95%-CI
Weight
finn-b-C3_COLON_ADENO_EXALLC 0.9560 [0.8855; 1.0321] 6.0%
finn-b-C3_COLON_EXALLC 0.9597 [0.8972; 1.0267] 7.5%
ukb-d-C18 0.9997 [0.9993; 1.0000] 43.3%
ukb-d-C3_COLON 0.9998 [0.9994; 1.0002] 43.3%
Random effects model 0.9940 [0.9741; 1.0143] 100.0%
Prediction interval [0.9168; 1.0777]
Heterogeneity: I^2 = 0%, τ^2 = 0.0002, p = 0.41
0.8 0.9 1 1.1 1.2
rs17050272

GWAS ID	OR	95%-CI	Weight
finn-b-C3_COLON_ADENO_EXALLC	0.9991	[0.9157; 1.0902]	0.0%
finn-b-C3_COLON_EXALLC	0.9943	[0.9208; 1.0737]	0.0%
ukb-d-C18	0.9999	[0.9995; 1.0004]	49.7%
ukb-d-C3_COLON	1.0000	[0.9996; 1.0004]	50.3%
**Random effects model**	**1.0000**	**[0.9996; 1.0004]**	**100.0%**
**Prediction interval**		**[0.9987; 1.0012]**	

Heterogeneity: $I^2 = 0\%$, $\tau^2 < 0.0001$, $p = 0.99$

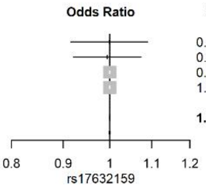

GWAS ID	OR	95%-CI	Weight
finn-b-C3_COLON_ADENO_EXALLC	0.9846	[0.9122; 1.0628]	0.3%
finn-b-C3_COLON_EXALLC	0.9831	[0.9190; 1.0517]	0.3%
ukb-d-C18	1.0000	[0.9996; 1.0004]	49.7%
ukb-d-C3_COLON	1.0000	[0.9997; 1.0004]	49.7%
**Random effects model**	**0.9999**	**[0.9960; 1.0039]**	**100.0%**
**Prediction interval**		**[0.9850; 1.0150]**	

Heterogeneity: $I^2 = 0\%$, $\tau^2 < 0.0001$, $p = 0.93$

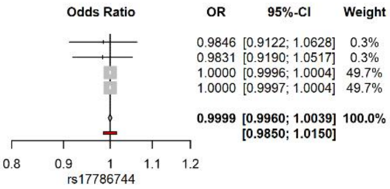

Supplementary Figure S4 Forest plot of the correlation between SNPs and rectal cancerr.

GWAS: genome-wide association study; OR: odds ratio; CI: confidence interval.

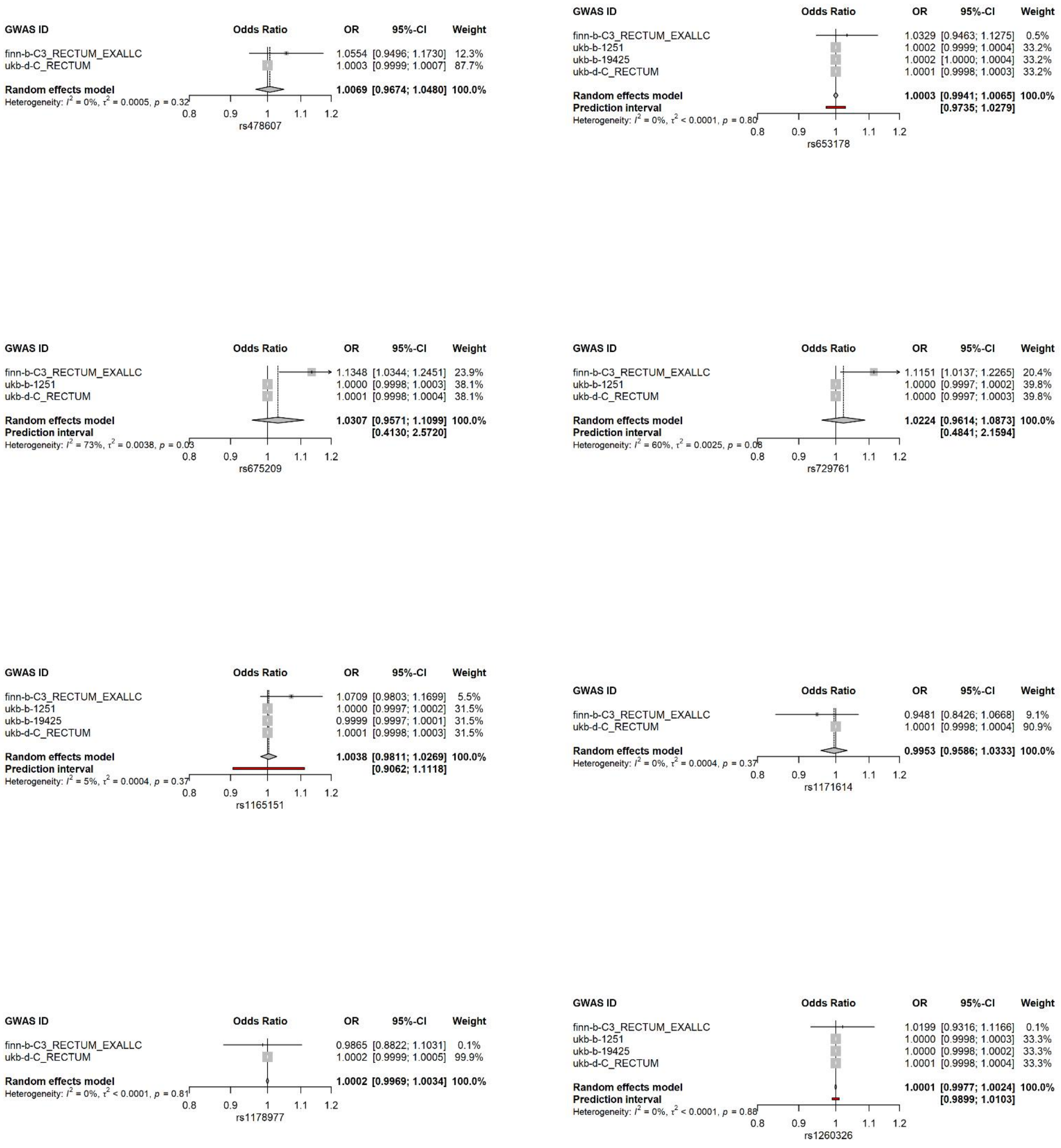

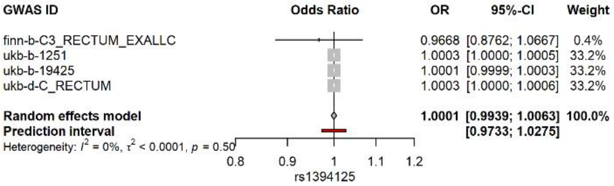
GWAS ID
Odds Ratio
OR
95%-CI
Weight
finn-b-C3_RECTUM_EXALLC 0.9668 [0.8762; 1.0667] 0.4%
ukb-b-1251 1.0003 [1.0000; 1.0005] 33.2%
ukb-b-19425 1.0001 [0.9999; 1.0003] 33.2%
ukb-d-C_RECTUM 1.0003 [1.0000; 1.0006] 33.2%
Random effects model 1.0001 [0.9939; 1.0063] 100.0%
Prediction interval [0.9733; 1.0275]
Heterogeneity: I^2 = 0%, τ^2 < 0.0001, p = 0.50
0.8
0.9
1
1.1
1.2
rs1394125

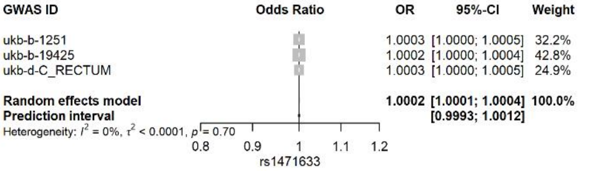
GWAS ID
Odds Ratio
OR
95%-CI
Weight
ukb-b-1251 1.0003 [1.0000; 1.0005] 32.2%
ukb-b-19425 1.0002 [1.0000; 1.0004] 42.8%
ukb-d-C_RECTUM 1.0003 [1.0000; 1.0005] 24.9%
Random effects model 1.0002 [1.0001; 1.0004] 100.0%
Prediction interval [0.9993; 1.0012]
Heterogeneity: I^2 = 0%, τ^2 < 0.0001, p = 0.70
0.8
0.9
1
1.1
1.2
rs1471633

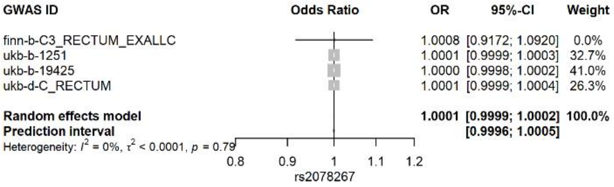
GWAS ID
Odds Ratio
OR
95%-CI
Weight
finn-b-C3_RECTUM_EXALLC 1.0008 [0.9172; 1.0920] 0.0%
ukb-b-1251 1.0001 [0.9999; 1.0003] 32.7%
ukb-b-19425 1.0000 [0.9998; 1.0002] 41.0%
ukb-d-C_RECTUM 1.0001 [0.9999; 1.0004] 26.3%
Random effects model 1.0001 [0.9999; 1.0002] 100.0%
Prediction interval [0.9996; 1.0005]
Heterogeneity: I^2 = 0%, τ^2 < 0.0001, p = 0.79
0.8
0.9
1
1.1
1.2
rs2078267

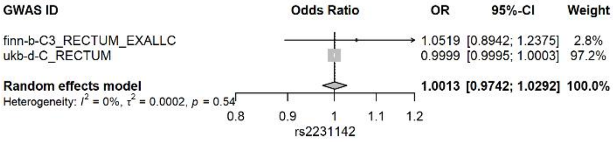
GWAS ID
Odds Ratio
OR
95%-CI
Weight
finn-b-C3_RECTUM_EXALLC 1.0519 [0.8942; 1.2375] 2.8%
ukb-d-C_RECTUM 0.9999 [0.9995; 1.0003] 97.2%
Random effects model 1.0013 [0.9742; 1.0292] 100.0%
Heterogeneity: I^2 = 0%, τ^2 = 0.0002, p = 0.54
0.8
0.9
1
1.1
1.2
rs2231142

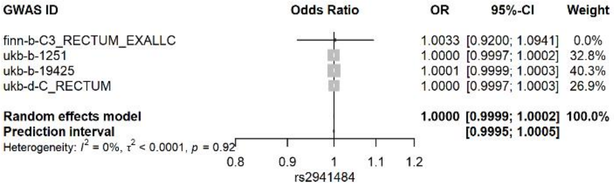
GWAS ID
Odds Ratio
OR
95%-CI
Weight
finn-b-C3_RECTUM_EXALLC 1.0033 [0.9200; 1.0941] 0.0%
ukb-b-1251 1.0000 [0.9997; 1.0002] 32.8%
ukb-b-19425 1.0001 [0.9999; 1.0003] 40.3%
ukb-d-C_RECTUM 1.0000 [0.9997; 1.0003] 26.9%
Random effects model 1.0000 [0.9999; 1.0002] 100.0%
Prediction interval [0.9995; 1.0005]
Heterogeneity: I^2 = 0%, τ^2 < 0.0001, p = 0.92
0.8
0.9
1
1.1
1.2
rs2941484

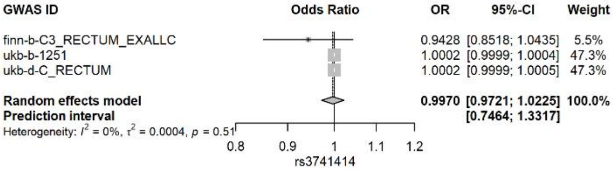
GWAS ID
Odds Ratio
OR
95%-CI
Weight
finn-b-C3_RECTUM_EXALLC 0.9428 [0.8518; 1.0435] 5.5%
ukb-b-1251 1.0002 [0.9999; 1.0004] 47.3%
ukb-d-C_RECTUM 1.0002 [0.9999; 1.0005] 47.3%
Random effects model 0.9970 [0.9721; 1.0225] 100.0%
Prediction interval [0.7464; 1.3317]
Heterogeneity: I^2 = 0%, τ^2 = 0.0004, p = 0.51
0.8
0.9
1
1.1
1.2
rs3741414

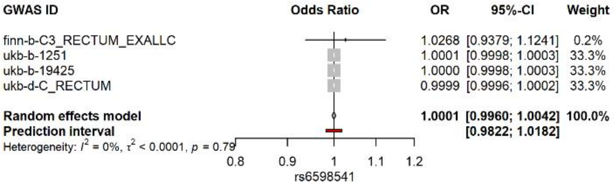
GWAS ID
Odds Ratio
OR
95%-CI
Weight
finn-b-C3_RECTUM_EXALLC 1.0268 [0.9379; 1.1241] 0.2%
ukb-b-1251 1.0001 [0.9998; 1.0003] 33.3%
ukb-b-19425 1.0000 [0.9998; 1.0003] 33.3%
ukb-d-C_RECTUM 0.9999 [0.9996; 1.0002] 33.3%
Random effects model 1.0001 [0.9960; 1.0042] 100.0%
Prediction interval [0.9822; 1.0182]
Heterogeneity: I^2 = 0%, τ^2 < 0.0001, p = 0.79
0.8
0.9
1
1.1
1.2
rs6598541

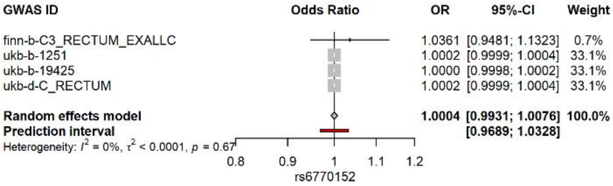
GWAS ID
Odds Ratio
OR
95%-CI
Weight
finn-b-C3_RECTUM_EXALLC 1.0361 [0.9481; 1.1323] 0.7%
ukb-b-1251 1.0002 [0.9999; 1.0004] 33.1%
ukb-b-19425 1.0000 [0.9998; 1.0002] 33.1%
ukb-d-C_RECTUM 1.0002 [0.9999; 1.0004] 33.1%
Random effects model 1.0004 [0.9931; 1.0076] 100.0%
Prediction interval [0.9689; 1.0328]
Heterogeneity: I^2 = 0%, τ^2 < 0.0001, p = 0.67
0.8
0.9
1
1.1
1.2
rs6770152

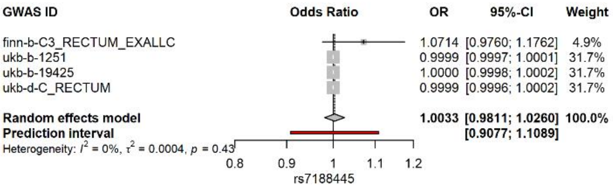
GWAS ID
Odds Ratio
OR
95%-CI
Weight
finn-b-C3_RECTUM_EXALLC 1.0714 [0.9760; 1.1762] 4.9%
ukb-b-1251 0.9999 [0.9997; 1.0001] 31.7%
ukb-b-19425 1.0000 [0.9998; 1.0002] 31.7%
ukb-d-C_RECTUM 0.9999 [0.9996; 1.0002] 31.7%
Random effects model 1.0033 [0.9811; 1.0260] 100.0%
Prediction interval [0.9077; 1.1089]
Heterogeneity: I2 = 0%, τ2 = 0.0004, p = 0.43
0.8 0.9 1 1.1 1.2
rs7188445

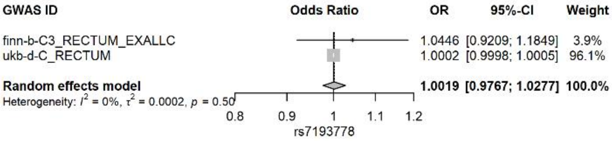
GWAS ID
Odds Ratio
OR
95%-CI
Weight
finn-b-C3_RECTUM_EXALLC 1.0446 [0.9209; 1.1849] 3.9%
ukb-d-C_RECTUM 1.0002 [0.9998; 1.0005] 96.1%
Random effects model 1.0019 [0.9767; 1.0277] 100.0%
Heterogeneity: I2 = 0%, τ2 = 0.0002, p = 0.50
0.8 0.9 1 1.1 1.2
rs7193778

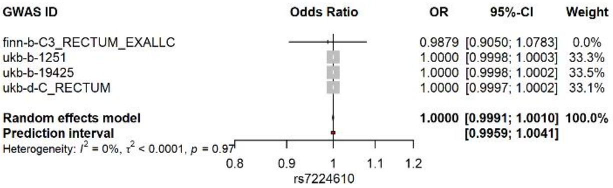
GWAS ID
Odds Ratio
OR
95%-CI
Weight
finn-b-C3_RECTUM_EXALLC 0.9879 [0.9050; 1.0783] 0.0%
ukb-b-1251 1.0000 [0.9998; 1.0003] 33.3%
ukb-b-19425 1.0000 [0.9998; 1.0002] 33.5%
ukb-d-C_RECTUM 1.0000 [0.9997; 1.0002] 33.1%
Random effects model 1.0000 [0.9991; 1.0010] 100.0%
Prediction interval [0.9959; 1.0041]
Heterogeneity: I2 = 0%, τ2 < 0.0001, p = 0.97
0.8 0.9 1 1.1 1.2
rs7224610

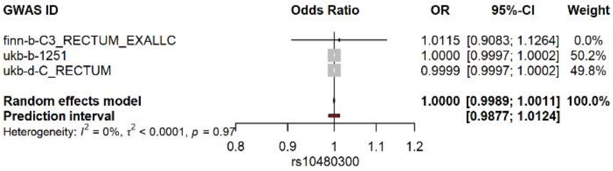
GWAS ID
Odds Ratio
OR
95%-CI
Weight
finn-b-C3_RECTUM_EXALLC 1.0115 [0.9083; 1.1264] 0.0%
ukb-b-1251 1.0000 [0.9997; 1.0002] 50.2%
ukb-d-C_RECTUM 0.9999 [0.9997; 1.0002] 49.8%
Random effects model 1.0000 [0.9989; 1.0011] 100.0%
Prediction interval [0.9877; 1.0124]
Heterogeneity: I2 = 0%, τ2 < 0.0001, p = 0.97
0.8 0.9 1 1.1 1.2
rs10480300

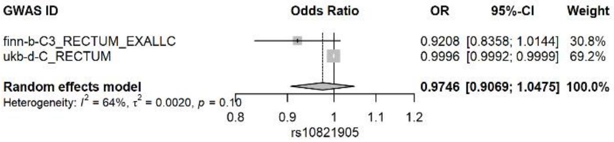
GWAS ID
Odds Ratio
OR
95%-CI
Weight
finn-b-C3_RECTUM_EXALLC 0.9208 [0.8358; 1.0144] 30.8%
ukb-d-C_RECTUM 0.9996 [0.9992; 0.9999] 69.2%
Random effects model 0.9746 [0.9069; 1.0475] 100.0%
Heterogeneity: I2 = 64%, τ2 = 0.0020, p = 0.10
0.8 0.9 1 1.1 1.2
rs10821905

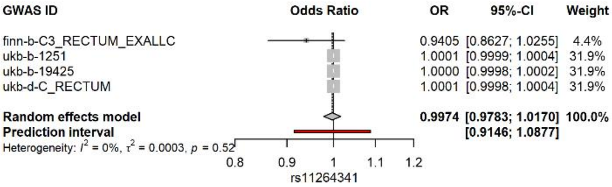
GWAS ID
Odds Ratio
OR
95%-CI
Weight
finn-b-C3_RECTUM_EXALLC 0.9405 [0.8627; 1.0255] 4.4%
ukb-b-1251 1.0001 [0.9999; 1.0004] 31.9%
ukb-b-19425 1.0000 [0.9998; 1.0002] 31.9%
ukb-d-C_RECTUM 1.0001 [0.9998; 1.0004] 31.9%
Random effects model 0.9974 [0.9783; 1.0170] 100.0%
Prediction interval [0.9146; 1.0877]
Heterogeneity: I2 = 0%, τ2 = 0.0003, p = 0.52
0.8 0.9 1 1.1 1.2
rs11264341

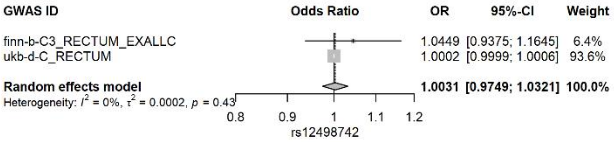
GWAS ID
Odds Ratio
OR
95%-CI
Weight
finn-b-C3_RECTUM_EXALLC 1.0449 [0.9375; 1.1645] 6.4%
ukb-d-C_RECTUM 1.0002 [0.9999; 1.0006] 93.6%
Random effects model 1.0031 [0.9749; 1.0321] 100.0%
Heterogeneity: I2 = 0%, τ2 = 0.0002, p = 0.43
0.8 0.9 1 1.1 1.2
rs12498742

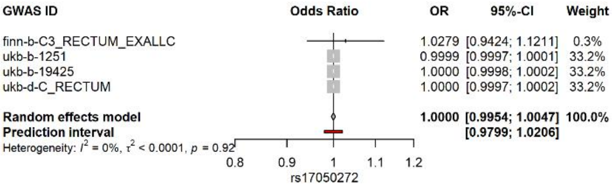
GWAS ID
Odds Ratio
OR
95%-CI
Weight
finn-b-C3_RECTUM_EXALLC 1.0279 [0.9424; 1.1211] 0.3%
ukb-b-1251 0.9999 [0.9997; 1.0001] 33.2%
ukb-b-19425 1.0000 [0.9998; 1.0002] 33.2%
ukb-d-C_RECTUM 1.0000 [0.9997; 1.0002] 33.2%
Random effects model 1.0000 [0.9954; 1.0047] 100.0%
Prediction interval [0.9799; 1.0206]
Heterogeneity: I2 = 0%, τ2 < 0.0001, p = 0.92
0.8 0.9 1 1.1 1.2
rs17050272

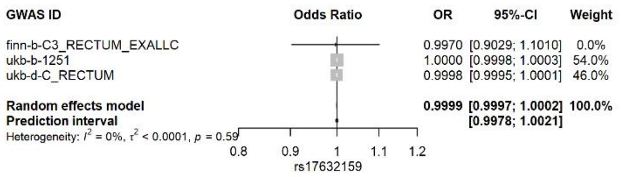
GWAS ID
Odds Ratio
OR
95%-CI
Weight
finn-b-C3_RECTUM_EXALLC 0.9970 [0.9029; 1.1010] 0.0%
ukb-b-1251 1.0000 [0.9998; 1.0003] 54.0%
ukb-d-C_RECTUM 0.9998 [0.9995; 1.0001] 46.0%
Random effects model 0.9999 [0.9997; 1.0002] 100.0%
Prediction interval [0.9978; 1.0021]
Heterogeneity: I^2 = 0%, τ^2 < 0.0001, p = 0.59
0.8 0.9 1 1.1 1.2
rs17632159

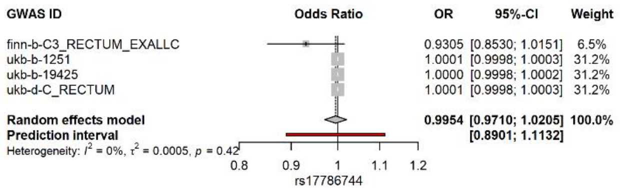
GWAS ID
Odds Ratio
OR
95%-CI
Weight
finn-b-C3_RECTUM_EXALLC 0.9305 [0.8530; 1.0151] 6.5%
ukb-b-1251 1.0001 [0.9998; 1.0003] 31.2%
ukb-b-19425 1.0000 [0.9998; 1.0002] 31.2%
ukb-d-C_RECTUM 1.0001 [0.9998; 1.0003] 31.2%
Random effects model 0.9954 [0.9710; 1.0205] 100.0%
Prediction interval [0.8901; 1.1132]
Heterogeneity: I^2 = 0%, τ^2 = 0.0005, p = 0.42
0.8 0.9 1 1.1 1.2
rs17786744
